# Substance abuse prevention infrastructure: a survey-based study of the organizational structure and function of the D.A.R.E. program

**DOI:** 10.1186/1747-597X-1-25

**Published:** 2006-09-06

**Authors:** Jeffrey C Merrill, Ilana Pinsky, Ley A Killeya-Jones, Zili Sloboda, Tracey Dilascio

**Affiliations:** 1 Division of Addiction Psychiatry, UMDNJ – Robert Wood Johnson Medical School, 671 Hoes Lane, Piscataway, NJ, USA; 2 Department of Psychiatry, R. Prof. Henrique Neves Lefevre, 71 casa 4, São Paulo – SP CEP-04637-000, Brazil; 3 Center for Child and Family Policy, Duke University, Box 90545, Durham, NC 27708–0545, USA; 4 Institute of Health and Social Policy, University of Akron, Akron, Ohio, 44325–1915, USA

## Abstract

**Background:**

The only national drug abuse prevention delivery system that supports the rapid diffusion of new prevention strategies and includes uniform training and credentialing of instructors who are monitored for quality implementation of prevention programming is the Drug Abuse Resistance Education network (D.A.R.E.) linking community law enforcement to schools. Analysis of the organizational structure and function of D.A.R.E. provides an understanding of the essential parameters of this successful delivery system that can be used in the development of other types of national infrastructures for community-based prevention services. Information regarding organizational structure and function around funding issues, training, quality control and community relationships was gathered through telephone surveys with 50 state D.A.R.E. coordinators (including two major cities), focus groups with local D.A.R.E. officers and mentors, and interviews with national D.A.R.E. office staff.

**Results:**

The surveys helped identify several strengths inherent in the D.A.R.E. program necessary for building a prevention infrastructure, including a well-defined organizational focus (D.A.R.E. America), uniform training and means for rapid dissemination (through its organized training structure), continuing education mechanisms (through the state and national conference and website), mechanisms for program monitoring and fidelity of implementation (formal and informal), branding and, for several states, predictable and consistent financing. Weaknesses of the program as currently structured include unstable funding and the failure to incorporate components for the continual upgrading of curricula reflecting research evidence and "principles of prevention".

**Conclusion:**

The D.A.R.E. organization and service delivery network provides a framework for the rapid dissemination of evidence-based prevention strategies. The major strength of D.A.R.E. is its natural affiliation to local law enforcement agencies through state coordinators. Through these affiliations, it has been possible for D.A.R.E. to become established nationally within a few years and internationally within a decade. Understanding how this structure developed and currently functions provides insights into how other such delivery systems could be developed.

## Background

Substance abuse brings with it a diverse array of human problems in every sphere of life, including decreased health, increased mortality, familial and social dysfunction, impaired educational and vocational opportunities, and increased involvement with the criminal justice system. It also leads to a cycle of abuse, perpetuating these problems and revisiting them on subsequent generations [1-7]. Despite the extent and gravity of these issues, substance abuse prevention efforts, that could reduce or avert many of these harms, are not significantly funded in the US. Of every dollar that American states spend annually on substance abuse, only 3.7¢ goes towards prevention, treatment, and research aimed at reducing the incidence and consequences of substance abuse [8]. Yet, an infusion of dollars into substance abuse prevention programs would likely lead to savings of thousands or even millions of dollars annually in healthcare, criminal justice, and child welfare system costs, and in regained work productivity [5,8-10].

In addition to insufficient funding, the field of substance abuse prevention lacks the organizational framework through which prevention services could be efficiently offered, organized, delivered, and paid for. Without such an infrastructure, assuring quality, monitoring performance, and rapidly and accurately diffusing new ideas and technologies, is very difficult. Many society-wide institutions – such as medicine, education, and the criminal justice system – have their own well-developed infrastructure that provides an array of services including advocacy, funding, innovation, dissemination, quality assurance, and accreditation and certification. For substance abuse prevention, however, there is no such infrastructure. The current ad hoc system of agencies, programs, curricula and activities is one that is under-funded, fragmented and lacking a central organizing body. This system lacks the ability or authority to accomplish necessarily complex tasks in an organized and professional manner. The current lack of organization of prevention services at any level of society limits the field of substance abuse prevention to efficiently serve the needs of communities, schools, parents and children.

Notwithstanding the lack of a formal substance abuse prevention infrastructure, there are numerous national and state-level organizations and entities that service some of the needs of such an infrastructure, including funding prevention activities and research, providing accreditation and certification, providing mechanisms for quality assurance and control, and disseminating information. However, even as some of these services are provided, they are fragmented. Furthermore, the lack of stable funding remains a significant problem as most prevention programming is dependent on government support, most often as a discretionary item and competing with other types of programs [5,11].

Federal entities such as the Department of Education with its Safe and Drug Free Communities program (SDFS) provides grant support to schools to implement prevention programs, the National Institute on Drug Abuse (NIDA) to support a national research program that evaluates innovative prevention strategies and on the basis of this research has developed its principles of prevention, the Center for Substance Abuse Prevention (CSAP) works to disseminate information about effective prevention strategies and policies and to assist local communities to decide what are most relevant to their needs. State departments or divisions of alcohol and drug abuse/addiction services as well as governors' offices also provide support for local prevention programming including credentialing of prevention specialists. Finally, to some degree, private foundations such as the Robert Wood Johnson Foundation (RWJF) support prevention and treatment service delivery. In addition, since the late 1990s, with recognition of the contributions being made by prevention research, [12-15] more and more funding agencies are demanding evidence-based prevention programming while, at the same time there is no clear definition or set of criteria that determine what "evidence-based" means [16]. Thus, in the field of substance abuse prevention, there is a disparate array of organizations, funding streams, and even definitions as to what effective prevention means. Therefore without some overarching unifying framework that could coordinate these efforts for greater synergy, prevention programming at the community level will continue to be fragmented and to suffer from the instability of funding and lack of professionalization of prevention as a field

With all of the promising prevention intervention programs that are currently funded, few pay much attention to the obvious need for developing or expanding an infrastructure to support a prevention service delivery system. What are the characteristics of such an infrastructure? In order to specify infrastructure characteristics it is important to state the aims of a prevention service delivery system. Such a system would prevent the use of tobacco, alcohol, marijuana and other drugs or delay the initiation of use of tobacco and alcohol among those who have not initiated use and reduce or eliminate existing use of these substances among those who initiated use. These aims would be achieved through the delivery of prevention strategies with proven effectiveness by well-trained instructors who implement the prevention strategy with fidelity. Prevention programming would be sustained over time. To achieve these ends, a supportive infrastructure would need to include a national professional organization with ties to local communities that would establish guidelines for the position of prevention specialist and for prevention programming. These guidelines would address ongoing training, monitoring, and technical assistance of credentialed instructors and the types of acceptable prevention approaches. The organization would need to be flexible to revise these guidelines in response to new knowledge about more effective delivery strategies. Public support and recognition of the importance of prevention programming would also need to be addressed as part of the infrastructure organization. Finally, there needs to be assurance that there will be ongoing funding for these services both nationally as well as at the local level from diverse sources including third party payers.

The only national delivery system for drug abuse prevention in the United States that comes closest to an ideal model is Drug Abuse Resistance Education or D.A.R.E. Essential to understanding how important D.A.R.E. is to the field of prevention is recognizing that this program has two important components, the delivery system itself, made up of thousands of trained local law enforcement officer-instructors, and the curricula that these officer-instructors present to students. This paper will focus on the delivery system and describes the structure, organization and function of D.A.R.E. Prior articles on D.A.R.E. focused on evaluating the short- and long-term impact of the curricula, most often the curriculum offered to elementary students in the 5^th ^or 6^th ^grade [18]. Those studies were important to the field in that they demonstrated the need to offer booster prevention interventions at the time that students enter the at-risk years when they are in middle school and high school [19]. The present investigation does not intend to discuss the effectiveness of its prevention program, but addresses, instead, the infrastructure components of its delivery system in terms of organization, communications, training, quality assurance, linkages to the broader prevention system, and funding. By describing the D.A.R.E. delivery system that has effectively spread throughout the United States and abroad, we hope to provide important information and insights as to how a national prevention infrastructure can evolve.

In its twenty years of operations, D.A.R.E. has developed into a national program involving every American state, operating in more than 1,800 school districts with over 15,000 police officers working in 8,300 schools [17,20]. Internationally, D.A.R.E. operates in more than 50 countries [17]. However, questions about the effectiveness of D.A.R.E. curricula (together with the threat of losing essential federal funds from SDFS) on the one hand, and acknowledgment of its unique network on the other led to a collaboration initiated in 1999 between D.A.R.E. program leaders, the University of Akron and The Robert Wood Johnson Foundation. The main objective of this collaboration has been to develop and test a new multi-level school-based substance abuse prevention program that, if demonstrated to have positive outcomes, will be both disseminated and delivered by the D.A.R.E. domestic and international network, and will maintain the use of D.A.R.E. officers as in-class teachers. An important element of this undertaking was the need to assess the utility of the D.A.R.E. delivery system. Under a subcontract from RWJF and University of Akron, researchers at University of Medicine and Dentistry of New Jersey (UMDNJ) set out to document and describe the organizational structure and function of the current D.A.R.E. operation within the United States.

Although there are numerous studies evaluating the effectiveness of D.A.R.E.'s curricula, to date there has been only one comprehensive assessment of the organizational structure and operation of the D.A.R.E. program, by the Research Triangle Institute (RTI) in 1993 [21], under a grant from the National Institute of Justice. The complete report remains unpublished, but a meta-analysis of D.A.R.E. evaluations included in the report was later published in the American Journal of Public Health[22]. The data collected for the RTI report although thorough is not timely in terms of D.A.R.E.'s current operations and procedures as the national leadership has changed since that time and reorganization has taken place. The present study was therefore initiated to examine the organization, functions, and monitoring procedures, and the flow of information and resources of D.A.R.E. across national, state, and local levels. Note that we did not plan an assessment of the utility or value of the current D.A.R.E. curriculum, nor did we make assumptions about what substance abuse prevention curricula might be used in the future. Rather, we asked whether the organizational structure of D.A.R.E. has the potential to serve as a model for informing the development of a substance abuse prevention system.

In the current paper, we report the results of interviews with D.A.R.E. America leadership, with all 50 D.A.R.E. state coordinators and two city coordinators (Washington DC and New York City have stand-alone D.A.R.E. programs) as well as the results of two focus groups held with D.A.R.E. officers. We use this information to illustrate the role of the state programs (from this point forward, "state programs" includes all states and Washington, DC and New York City), the relationship between the states and local initiatives, and the relationship between the states and the ostensible organizing operation at D.A.R.E. America.

## Results

**Figure 1:**
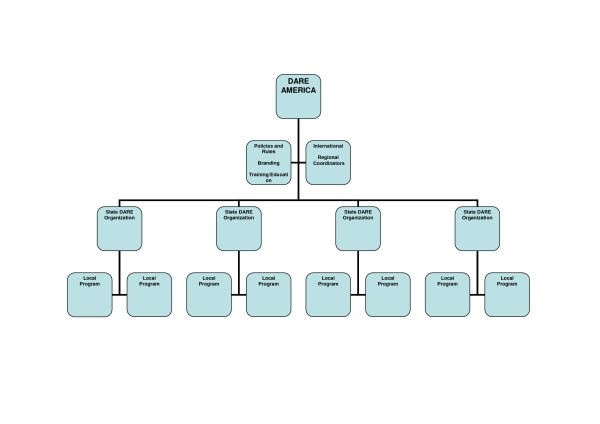
Organization, structure and function of DARE program in the United States.

The interviews provided an outline of the D.A.R.E. organizational structure which consists of an umbrella group, D.A.R.E. America that is located in Los Angeles and a network of local, grassroots law enforcement agencies (See Figure 1). State level organizations serve to support the local groups and to communicate between them and D.A.R.E. America. To foster additional communication between the state and D.A.R.E. America are seven Regional Coordinators.

### Structure and operation of D.A.R.E. America–the national organization

D.A.R.E. America's "corporate structure" is comprised of a president and several directors, including those responsible for communications, Charter Organizations, implementation and development of training, education, marketing, licensing and trademark enforcement, and international liaisons. There are also seven Regional Program Directors, each responsible for between five and seven states, who serve as the primary link between D.A.R.E. America and the states. D.A.R.E. America operates with the assistance and guidance of four advisory groups, each of which provides input and advice about a specific aspect of D.A.R.E.'s program and mission, including education, law enforcement, science, and youth.

D.A.R.E. America establishes national policies that define the rules of conduct, procedures for formal agreements to be established between schools and law enforcement organizations around the delivery of school curricula, criteria for selecting D.A.R.E. officers, mentors and training facilitators, requirements for training and maintenance of proficiency, rules regarding decertifying/suspending a D.A.R.E. officer, and how to handle student disclosures. The national office also establishes uniform methods for training, including on-line training, and certification, and controls the distribution of both instructor and student materials that bear the D.A.R.E. logo and trademark. Even D.A.R.E. vehicles, often donated by local area car dealerships must be registered, can display only the D.A.R.E. logo and can be used only for D.A.R.E.-associated business.

Another important function of the national D.A.R.E. organization is to facilitate communication across the various levels of the nationwide network and between the diverse stakeholders, such as state coordinators, D.A.R.E. officers, school administrators, classroom teachers, parents, students and concerned others. There are numerous channels used for maintaining such communication, including the D.A.R.E. website [23], educational materials and curricula, the annual national training conference, state in-service trainings, and D.A.R.E. start-up kits of materials and curricula for new or restarted local-level programs. Of particular importance to the D.A.R.E. officers who deliver the prevention education programs is the annual National D.A.R.E. Officers Association Conference, open to all D.A.R.E. officers and educators. These conferences serve as a major information dissemination and training vehicle and primary forum for the introduction of new approaches to prevention interventions. It was noted in an earlier descriptive study of D.A.R.E. officers [24], that because of their prevention role, officers feel detached and isolated from their agency colleagues who are more focused on law enforcement per se. The annual conference therefore serves to reinforce the special connection that D.A.R.E. officers need to maintain their identity and their commitment to their prevention role. Finally, the conference provides another opportunity for maintaining the integrity of the D.A.R.E. logo and symbol.

A relatively new component of the D.A.R.E. system is that of Charter Organizations, not-for-profit 501(c) (3) entities structured at the state level. To date, 26 states are chartered with a number of others moving in this direction. The emergence of Charter Organizations represents an effort to tighten the organization and to better define the roles of the various levels. They are intended to assure more uniformity of curriculum, of organizational structure in each state, and of program monitoring. Chartered states are required to file annual reports with D.A.R.E. on their activities and financial condition, permitting D.A.R.E. America to monitor state activity, provide assistance where needed and, in the case of a major breach on the part of the state (e.g., where it ignores the D.A.R.E. curriculum), rescind the Charter. While such Chartered Organizations do not receive services or benefits over and above those available to non-chartered states, the objective is to formalize such arrangements and strengthen the D.A.R.E. organization as a nationwide entity with the addition of a level of more structured oversight and control

### Structure and operation of state level D.A.R.E. organizations

The features discussed in this section include organization, funding, training and quality control, community efforts, and the issues and concerns elaborated by the state coordinators.

#### Organization

Although there is no single organizational model at the state level, 44 state networks are affiliated with government agencies in the law enforcement sector while the remaining six are run by private, not-for-profit organizations. The diversity of affiliations for the state-level organizations is probably the result of their grass-roots, bottom-up evolution that met the specific needs and organizational relationships that already existed. For example, overall, the structure for the state-level groups includes a coordinator, the state D.A.R.E. Officers Association and its board, mentors, and D.A.R.E. officers

Each state D.A.R.E. organization has a coordinator who fulfills a number of administrative and programmatic functions, including administering the state network of D.A.R.E. officers; liaising between D.A.R.E. and other state agencies, legislature, media, and local police departments; planning and executing training activities; monitoring program fidelity and quality control; and fund-raising. As befits the location of the majority of state organizations many (forty) coordinators are active or retired members of law enforcement agencies. They have a wide range of experience in the coordinator position, from just a few months to 14 years; the average time served is five years while the mode is four years.

The ways in which the coordinators spend their time depends on a number of factors, including percentage of full-time effort dedicated to the coordinator position, the funding level of the state organization, and the size and independence of the local police departments involved with the D.A.R.E. Twenty-two coordinators dedicate 80–100% of their time, while twenty dedicate less than 50% This variation in percentage of time devoted to the state-level D.A.R.E. operation is a function of the available resources, as funding levels vary widely across state organizations, and/or the needs of the program in any given state. Funding levels also impact the availability of support staff, thus constraining the extent of the coordinators' efforts.

A number of the coordinators also divide their time between the state and the local levels, giving them greater opportunity to participate in local activities. D.A.R.E., when viewed from the local level, is still fundamentally a grass roots organization that is, to a great degree, autonomous, operating independently not only of the state but also of the national organization. The ways in which D.A.R.E. is implemented and supported in any given community and even the extent to which it is implemented across a particular state, is ultimately predicated upon loose-knit agreements between the local police departments, schools, and parents. Moreover, although some local programs apply for the U.S. Department of Education Safe and Drug Free Schools program funding through their state organizations, many cannot rely upon the state D.A.R.E. organization for funding. Therefore, the state organization and by extension, the state coordinator, has limited influence over constituent local programs, including how many officers are trained, whether they are full-time, or even choosing which officers will become D.A.R.E. officer-instructor candidates.

The state coordinators have more extensive involvement at the local level with training, monitoring and quality control functions. These functions include evaluation of the delivery of the curricula providing feedback to the officers on their teaching methods. In addition, fifty percent of state coordinators are voting members on the board of their state's D.A.R.E. Officers Association. The state D.A.R.E. Officers Associations (DOA) are private and represent the interests of the local police force membership who are the D.A.R.E. officer-instructors. The position of the state coordinator on the state DOA board confers voting authority on state program issues, facilitates closer working relationships with the D.A.R.E. officers, and results in benefits for the state organization overall, particularly with respect to quality control, curricula implementation fidelity, organization of state trainings (that are often done in partnership with the state's DOA) and communication of updated information from D.A.R.E. America.

#### State-level funding

It is important to note that states do not have any formal financial relationship with D.A.R.E. America, even those with Charter status. To some extent, this fiscal independence informs the nature of the funding models for the state D.A.R.E. organizations which vary widely from state to state in terms of how funding is arranged, the number of funding sources, the amount that is dedicated to program delivery, and how funding is used. Table 1 shows that some states can rely on a variety of funding sources, whereas others depend upon just one. Seven states can be considered "millionaire" states, with more than a million dollars in the annual operating budget, while fourteen states report budgets of $50,000 or less. State funds principally are used for D.A.R.E. officer salaries, training costs, travel to state and national conferences, and teaching materials and supplies.

**Table 1:** Placement, sources of funds, and funding level (FY 2000) of state level D.A.R.E. programs.

State	Placement of D.A.R.E. Program^a^	Source(s) of Funds	Funding Level in $000s: FY 2000^b^
Alabama	Huntsville P.D.	Legislative: general revenue	B--$353
Alaska	State Troopers	Byrne Grants^c^, SDFS^d^, donations	A--$98
Arizona	AZ Public Safety	Grant (Dept. of Health Services), Donations	B--$217
Arkansas	Blytheville P.D./Criminal Justice Academy	Tuition^e^	A--$3
California	L.A.P.D.	D.A.R.E. America	C--$875
Colorado	Special Operations Bureau, Aurora	Fundraiser	B--$125
Connecticut	CT. Police Academy	Federal grant	A--$70
Delaware	State Police	Drug recovery/forfeiture	A--$30
Florida	FL Dept. of Law Enforcement	Legislative: general revenue	B--$393
Georgia	GA Bureau of Investigation	Legislative: general revenue	A--$60
Hawaii	Honolulu P.D.	SDFS	C--$100
Idaho	State Police	Byrne Grants	A--$30
Illinois	State Police	SDFS	C--$1,500
Indiana	Private Non-profit: Multiple Assc. Management	Fundraiser, Tuition	B-$125
Iowa	State Patrol	Dedicated (Surcharges), Tuition	A--$78
Kansas	Attorney General's Office	Legislative: general revenue	B--$160
Kentucky	State Police Academy	Legislative: general revenue	A--$60
Louisiana	Private Non-profit: Red River Delta Law Enforcement Planning	Legislative: general revenue	C--$3,900
Maine	Dept. of Public Safety	Tuition, Legislative: general Revenue	A--$13.5
Maryland	MD Police Corrections Training Commission	Legislative: general revenue	B--$200
Massachusetts	Governor's Alliance Against Drugs	Dedicated (Tobacco Tax), SDFS	C--$4,300
Michigan	School of Criminal Justice, Michigan State University	Byrne Grant, Tuition, Donation	C--$502
Minnesota	Private Non-profit: D.A.R.E. Minnesota	Fundraiser, Donations	B--$240
Mississippi	Lee County Sheriff's Office	Tuition, Byrne Grant	A--$60
Missouri	Dept. of Public Safety	Federal grant, Legislative: general revenue	C--$558
Montana	Dept. of Justice	Donation (United Way)	A--$4
Nebraska	State Patrol	SDFS	A--$45
Nevada	LV Metro PD	Tuition	A--$15
New Hampshire	State Police	Donation (Masons), Fundraiser	A--$58
New Jersey	Private Non-profit: D.A.R.E.NJ	Donations (recycling^f^), SDFS	C--$1,124
New Mexico	Silver City Police/County Sheriff's Office	Tuition, D.A.R.E. America	A--$16
New York	State Div. of Criminal Justice Services	Legislative: general revenue	B--$300
New York City	D.A.R.E. America	Corporate Support, Fundraisers	C--$1,000
North Carolina	State Bureau of Investigations	SDFS, Legislative: general Revenue	C--$614
North Dakota	Fargo PD	Local level solicitations	Unknown--mostly supported locally
Ohio	Private Non-profit: Law Enforcement Foundation	Dedicated (Surcharges), SDFS, Donation	C--$2,000
Oklahoma	Dept. of Public Safety	Byrne Grants	B--$197
Oregon	Dept. of Public Safety Standard Training	Dept. of Public Safety Standard Training	A--$20
Pennsylvania	PA Commission on Crime And Delinquency	Legislative: State budget line item, Donations	C--$5,200
Rhode Island	Newport P.D.	SDFS	A--$22
South Carolina	State Police	Legislative: general revenue	B--$200
South Dakota	Law Enforcement Training Academy	Dedicated: Surcharges	B--$100
Tennessee	Dept. of Safety	SDFS	A--$78
Texas	Center for Safe Communities And Schools	SDFS, Tuition	B--$400
Utah	Private Non-profit: Council for Crime Prevention	Legislative: general revenue, SDFS, Drug recovery/Forfeiture	A--$75
Vermont	Criminal Justice Training Council	SDFS, Fundraiser, Donations, Tuition	A--$27
Virginia	State Police	Donations, Legislative: general revenue, SDFS, Forfeiture	A--$222
Washington	Criminal Justice Training Commission	Awaiting new funding source	A--$170
Washington, DC	U.S. Park Police	Park Police Budget (Fed. Govt.)	A--$35
West Virginia	Charleston P.D.	Legislative: general revenue, Dedicated	Unknown at the time of survey
Wisconsin	Dept. of Justice	Drug recovery/Forfeiture	B--$100
Wyoming	Hot Springs County Sheriff's Office	State grant	A--$20

^a^Program locations are State or Local Government unless noted. ^b^Level of funding. A = less than $100,000; B = $100,000 – $500,000; C = more than $500,000. ^c^The Edward Byrne Memorial State and Local Law Enforcement Assistance Program, Department of Justice. ^d^Federal government Safe and Drug-Free Schools program. ^e^Tuition paid by police departments for training D.A.R.E. officers. ^f^Recycling refers to funds derived from local community clothes and toys recycling programs.

There are two major and well-established Federal funding mechanisms for state D.A.R.E. organizations, the SDFS and grants awarded under the aegis of the Byrne Amendment (The Edward Byrne Memorial State and Local Law Enforcement Assistance Program at the Department of Justice). In fiscal year 2000, the SDFS program provided approximately $439 million in grants to various schools and communities [22], of which some was used for D.A.R.E. or other school-based drug prevention curricula and programs. Table 2 summarizes the funding sources for state-level D.A.R.E. organizations.

**Table 2:** Summary of funding sources for D.A.R.E. programs

Funding Source	Examples	Number of States^a^
Federal Pass-through	SDFS^b^, Byrne Grants^c^	20
Legislated (State)	General Revenue, State Budget Line Item	16
Donations/Fund Raisers	Masons, United Way	13
Dedicated Funds	Surcharges, Tobacco Tax, Drug Recovery & Forfeiture	06
Other Federal & State Grants	Dept. Health Services	03
D.A.R.E.-related	Officer Tuition, D.A.R.E. America	11

^a^Includes Washington, DC and New York City programs. Column totals to more than 52 as programs often report more than one source of funding. ^b^Safe and Drug Free Schools program, Department of Education, now known as the Safe and Drug Free Schools and Communities program (SDFSC). ^c^The Edward Byrne Memorial State and Local Law Enforcement Assistance Program, Department of Justice.

The main source of funding for the day-to-day operations of the majority of the state organizations is the local law enforcement agencies, that often covers the D.A.R.E. officers' salaries (including overtime costs related to their D.A.R.E. activities), provides D.A.R.E. vehicles, and in many cases, pays the costs for police officers enrolled in the D.A.R.E. training program including associated travel expenses, meals, lodging, and supplies. These law enforcement agencies also cover the salaries of the D.A.R.E. mentors, discussed in more detail in the Training and Quality Control section below. D.A.R.E. America quantified the time and value of police officers (and volunteers) at $217 million for FY 2000 [25], an amount approximately 10 times what D.A.R.E. America receives from Federal government grants. However, others have suggested that this figure is inaccurate arguing that the real cost of using police officer time is closer to three times that amount particularly if one includes opportunity costs related to such items as classroom time that could be used for other activities and lost work time of officers going for D.A.R.E. training [26,27].

#### Training and quality control

The variation in funding discussed above is reflected in each state organization's training and quality control activities. Three important components of these activities are formal training programs, mentors, and quality control efforts.

#### Formal training programs

In fiscal year 2000, the Bureau of Justice Assistance provided more than $2 million for D.A.R.E. regional training centers to support the training of new officers [18]. With the recent reorganization of D.A.R.E., these centers were deactivated, placing the responsibility of the organizing and funding of training with each state's training center. The number and type of training activities available in different states varies considerably. D.A.R.E. offers four types of trainings for its officers: the CORE (a term no longer used by D.A.R.E.) program (typically used with 5^th ^and 6^th ^grade students), the Junior High, Senior High and Parent programs. CORE is an intensive 80-hour program given over two weeks. All officers wanting D.A.R.E. training must receive this more extensive "basic" training and have at least one year of experience with the elementary school curriculum before being trained in the other curricula. This basic course is generally offered by each state 1–2 times each year, but its annual periodicity ranged from 0 (North Dakota) to 12 (California).

Additional training and continuing education takes place at the national D.A.R.E. officers' conference and most states hold their own in-service training conferences each year, often in collaboration with the state D.A.R.E. Officers Association conferences. D.A.R.E. America directs the states to use these conferences to administer training for those unable to attend the national conference, and assists in the planning and coordination of these activities. In 2000, attendance at the state's annual conferences ranged from a low of 1% in New York City to a high of 100% in four states. The median attendance rate was 50%.

#### Mentors

Mentors are usually senior D.A.R.E. officers in the program who have extensive experience teaching the D.A.R.E. elementary school program. Across states, mentors vary in number, quality, and training. The number of mentors in each state ranges from 0 (in Delaware and North Dakota) to 30 (in New Jersey), with a median of eight, while the number of D.A.R.E. officers active in each state varies from 7 (in Delaware) to 1,250 (in New Jersey), with a median of 200. Thus, the ratio of mentors to officers varies from a high of 1: 5 in South Carolina, to a low of 1: 98 in Illinois, with a median of 1: 25. Mentors serve as a resource to the officers, are integral to the infrastructure of D.A.R.E. and the quality control process, provide both formal and informal instruction, and liaise between the officers, schools and state D.A.R.E. programs. Given the diversity and importance of these roles, mentors can mean the difference between more successful and less successful program delivery.

#### Quality control

We assigned states to one of three levels of quality control: formal, informal, or none, based on the levels of three indicators: periodicity of formal quality control visits made to the programs/curricula, use of standardized quality control forms to monitor performance and program adherence, and presence of designated persons to carry out quality control activities. A state's quality control system was considered formal if there was a preset periodicity of visits to the schools by designated personnel to monitor the in-class performance of the D.A.R.E. officers to assess their adherence to the D.A.R.E. curriculum and pedagogical goals, and if such assessment was supported by and facilitated with standardized forms. Other characteristics of formal quality control included the provision of feedback following monitoring and assessment activities and the use of focus groups to discuss and resolve problems and uncertainties. Informal quality control procedures included limited, random, ad hoc or problem-solving approaches to monitoring of officers with no set periodicity. The officers in these states then had little or no supervision, often receiving no feedback about their performance. Twenty states had formal quality control procedures in place while nineteen states (including the two city organizations) instituted varying levels of informal quality control procedures. The remaining thirteen states had no quality control procedures in place.

Overall, quality assessments were completed by observation. The composition of observation groups did vary across states. Half of those states with a formal quality control process in place used D.A.R.E. mentors only while 6 of the states had mentors and teachers do the observations; two of these states used teachers only; one, teachers and students; and one, a combination of a mentor, teachers and students. Thirteen of the states with informal quality control procedures included observations as an assessment tool. Ten of these states used a mentor system only while two used teachers only and two, a combination of mentors and teachers.

Thirty-two states used forms to rate the officers' in-class performance (including some of the states whose quality control activities were classified as informal, overall). Most states developed their own assessment forms. The majority (56%) of these forms focused on appropriate instructional style while the assessment of content coverage was included on the forms for about 25% of the states. Of the states with no apparent quality control procedures in place, three were in the process of developing them. However, six others reported that no such procedures would or could be initiated due to lack of funds, and two states that previously conducted quality control activities halted them when sufficient financial support could not be found.

Although there is some room for improvement in the extent and level of quality control, it is important to keep in mind that the D.A.R.E. organization seems to compare favorably to other current school-based substance abuse prevention programs that have been developed and received federal recognition, such as Project Alert and Life Skills Training. For example, through its website, Project Alert makes implementation assessment instruments available to its trained teachers [28], however there is no requirement that the information be collected or returned. Similarly, Life Skills Training's program, coordinated by National Health Promotion Association, Inc [29] provides but does not require its instructors to use their standardized implementation checklists. It should be pointed out that in general, unlike the D.A.R.E. assessment approach, most of these other assessments are self-administered by the instructors.

#### Community efforts

D.A.R.E. officers link to their communities at the local level in many ways, such as serving in other roles outside of the schools, as community organizers, facilitators, and referral sources to other programs and resources, all of which enrich and expand the officer's role [24]. In contrast, contacts between the state coordinators and other prevention programs in their communities were generally poor. Fully half of the state coordinators remarked that they had no relationships with other prevention programs in their communities. Of the coordinators that did report some contacts, nine said that such contacts were either informal or ad hoc, or consisted of attending rallies and manning booths at annual fairs. Thus, only 17 state coordinators reported any formal involvement with other nationally-recognized entities such as MAAD, SAAD, and Red Ribbon, or more local programs. Formal contact often occurred when the state coordinator also acted as the head of the prevention division in their state, or sat on the board of one of the local programs.

The D.A.R.E. officers were connected to the community in thirty-eight states through the D.A.R.E. Parent Education program. This program consists of five lessons designed to educate parents about drugs and about parenting. The D.A.R.E. officer guides discussion groups around a variety of topics. About a quarter of the state organizations were severely limited as to how many of these groups could be provided as only one or two D.A.R.E. officers were available for parent groups state-wide. Twelve states (as well as NYC and DC) had no parent program in place. Parental involvement and program support varied widely both within and between states, regardless of whether a fully functioning parent program was in place. Twenty-six (50%) state coordinators characterized parent involvement as low, and five (9%) reported that involvement varied by location but was often poor. Thirteen coordinators (25%) said that parent involvement in their states was good, and eight (15%) said that involvement was excellent. In general, then, parents overall are very enthusiastic about the program, but this warm feeling does not necessarily translate into participation in parent curriculum classes or attendance at other more formal activities.

#### Issues and concerns

We asked the coordinators to describe any issues or concerns they had about their relationship with the national organization or the D.A.R.E. network in general. Their responses were organized under the headings: funding, communication, training, program implementation fidelity, and the new curriculum in development. Many coordinators expressed concern over their funding. Some states were in the process of losing their traditional lines of funding and coordinators remarked that some form of national or uniform funding would be beneficial. Communication with D.A.R.E. America was sometimes viewed as poor, especially in terms of the changes in the organizational structure of training. Several coordinators expressed concerns with the fidelity of program implementation; as noted above, a number of states had discontinued their quality control procedures for lack of funds, and others reported that no such quality control procedure could be put in place without increased funds for its implementation and support. Lastly, there were concerns about how the impending new curriculum being evaluated by researchers at the University of Akron would be disseminated, fueled by a perceived lack of communication from D.A.R.E. America about the development and testing of the new curriculum.

## Discussion

This paper describes the D.A.R.E. national delivery network. The role of D.A.R.E. America has evolved from a local prevention delivery unit to an international diffusion and dissemination organization. It is through D.A.R.E. America that programs are developed, that national mass training protocols are designed, and that relationships are maintained with state and federal governmental agencies and national law enforcement organizations. Analysis of the network indicates that D.A.R.E. is not a monolithic organization, but is rather decentralized, with clearly differentiated roles, functions, funding, and financial management systems. D.A.R.E. America is the glue that holds together the network of local and state support organizations, and that connects grassroots level D.A.R.E. officers who actually go into the schools and deliver prevention programs, to the broader network of officers within the state and nationally. The state-level D.A.R.E. entities function to recruit and screen new D.A.R.E. officers, to administer local training of these officers and continuing education for all D.A.R.E. officers, to monitor the quality of program implementation, to maintain relationships with state and local governmental agencies, and to help solicit federal, state and local funding for training and mentoring.

It is important that the D.A.R.E. delivery network be seen as separate from its programming. Until 1999, D.A.R.E. America in conjunction with educators from the Los Angeles Unified School District developed prevention programs to be delivered in elementary, middle and high schools. These programs had undergone regular periodic revisions every few years, reflecting information from the research literature and the requests of local and national agencies. Local law enforcement agencies in conjunction with local school districts are the ones who decide which of the K though 12 programs offered by D.A.R.E. America will be delivered in the schools. Generally, these decisions have been influenced by the availability of manpower and funding.

Eighty percent of school districts in the United States offer D.A.R.E. programs, the vast majority of which are directed to elementary school children [20]. In fact, this is reflected by the amount of training offered in the various levels of curricula: while 92% of the states offered the D.A.R.E. Core program, directed at elementary school children, only 58% offered the junior high and 27% the senior high programs. With the new federal and, in many cases, state requirements for the delivery of evidence-based prevention programming, local D.A.R.E. organizations have been struggling to support their activities. In several cases, these organizations have opted not to deliver D.A.R.E. programs but have either adopted other programs that have demonstrated success or sought local rather than federal funding for their salaries, D.A.R.E. materials and other associated expenses. This is occurring at a time when D.A.R.E. America has committed to cooperate with the University of Akron study to evaluate a new evidence-based program directed to middle and high school students. When this evaluation is completed in 2006, the findings will guide D.A.R.E. America's future programming. If effective, the program could be rapidly disseminated throughout the United States and abroad within one to two years. However, even with the elaborate D.A.R.E. delivery network in place, there remain significant weaknesses that must be addressed. These have been identified in this paper and are discussed below under funding, training and quality assurance.

### Funding

There have been both advantages and disadvantages associated with the decentralization of funding for local D.A.R.E. efforts. By state and local organizations having the responsibility for funding on-going operations, there is more community control over these programs. On the other hand, when funding and manpower are scarce as is the current situation, many communities are eliminating the position of the D.A.R.E. officer and moving more officers onto the streets. The state D.A.R.E. organizations are competing with other social and health services agencies for a decreasing pool of available funds. In addition, those communities that depend on federal funds from the Department of Education's Safe and Drug-Free Communities are struggling to meet the Department's principles of prevention and are either attempting to evaluate their local programs or to seek waivers while waiting for the University of Akron's study to be completed. Clearly, there is a need to address the funding issue to establish continuity of prevention services in our nation's schools. At all levels, D.A.R.E. organizations are meeting with policy makers to maintain D.A.R.E. programming. Success of these meetings varies, depending on the stability of local police efforts. It can be seen by the results in this paper that, although funding is still a concern and many state programs struggle with this issue, some of the D.A.R.E. state programs seem to have found relatively stable and reasonable funding. In our interviews, we were introduced to several funding models, but the more successful ones seem to be those that combine several funding mechanisms, which require time, dedication and knowledge about fund-raising mechanisms on the part of the state-level coordinators.

### Training

Until recently, all training of new and retraining of existing D.A.R.E. officers had been carried out at regional training centers. The training itself was didactic, a strategy used in the earlier prevention curriculum design for students. At the time that D.A.R.E. America began its collaboration with the RWJF and The University of Akron, a commitment was made to revise its elementary, middle and high school curricula to reflect the latest prevention research findings. This research suggested important content elements and instructional strategies that engaged children in the learning process. These new approaches changed the instructor's role from lecturer to facilitator or coach. Such a dramatic change required major changes in training. D.A.R.E. America has therefore eliminated the regional training centers and designed a training-of-trainers approach that involved all of the state D.A.R.E. organizations' educators to conduct all the training within each state. The training is more 'hands on' than was the case before and includes additional training in classroom management and facilitation techniques. An evaluation of this new approach is in the planning stages.

### Quality assurance

One of the major positive features of the D.A.R.E. delivery network has been quality assurance. Mentors were trained to observe the delivery of programs within the classroom setting, to conduct debriefings with the classroom D.A.R.E. officer and to make recommendations for additional training or for dismissal. This study has found that the decentralization of D.A.R.E. weakens this organizational component. Despite the fact that most states had concerns about implementation quality, and many of them had formal forms of quality assurance in place, shortage of funding has limited the availability of manpower to make visits to all classrooms to monitor implementation. This is not solely a problem associated with D.A.R.E., as other prevention program models also do not incorporate ongoing monitoring of implementation fidelity. However, the frequency of quality assurance already in place and the concerns expressed by state coordinators and D.A.R.E. officers suggest recognition of the importance of ongoing evaluation of program delivery.

## Conclusion

D.A.R.E., the major national network that supports substance abuse prevention programming delivered in our schools is undergoing reorganization to meet the current needs of its constituents and the local D.A.R.E. officers. This is going on at a time when D.A.R.E. is responding to criticisms of its prevention curricula and when it is being challenged financially. Despite its several faults at the organization level, D.A.R.E. is an attractive program for communities as it possesses several characteristics necessary for building a prevention infrastructure. These include uniform training and through its organized training structure, the means for the rapid dissemination of updated prevention programming; through the state and national conferences and websites, continuing education; mechanisms for program monitoring and quality assurance; and models for predictable and consistent financing. As such D.A.R.E. remains as a model of a prevention service delivery system that has survived over twenty years. Key to its survival has been its decentralized structure, using law enforcement agencies as a platform for programming that allows local communities to function autonomously.

At the same time an overarching organizational structure has been developed to address the more global issues of development of evidence-based curricula, "accreditation" through the process of selecting and training of officer-instructors, the production of standardized program materials, having representation of all D.A.R.E. units at the national and international level, collecting and disseminating information pertinent to all D.A.R.E. officers and units, and, in some ways most important, control of the D.A.R.E. brand. The retirement of the founder and Chief Executive Officer, Glenn Levant, in 2003 has prompted a reorganization of D.A.R.E. America, a reassessment of its future direction, the building of new collaborations, and the restructuring of old networks. The fragility of funding, the requirement of evidence-based prevention curricula and adherence to "principles of prevention" has forced D.A.R.E. to face both its' strengths and weaknesses. If D.A.R.E. survives these new threats it could serve as a model prevention infrastructure that can be emulated by other national groups.

Several conclusions can be drawn from this analysis of the D.A.R.E. delivery system that would inform the development of an integrated national prevention service delivery system. Having a national umbrella organization that sets standards for the delivery of quality services allows control over the "brand" including

• the selection of the prevention programming strategies/services,

• development of manuals or guidelines for the delivery of the services,

• specifying the criteria and knowledge needed by prevention instructors,

• training in this knowledge base and how those services are to be delivered, and

• monitoring both the delivery and the outcomes of service delivery.

This centralized organization would serve as a conduit for the rapid dissemination of new information as improvements in service components and service delivery become available.

As essential as an umbrella organization is, there needs to be a supportive structure at the state and local levels. The success of the dissemination of the concept of "D.A.R.E." is most likely due to the interconnectedness of law enforcement agencies. Several national and state organizations of police chiefs and sheriffs exist and through these organizations' newsletters and conferences law enforcement administrators learn about new services and technologies. It was through these networks that the concept of D.A.R.E. disseminated so rapidly.

## Methods

Data for this study came from telephone surveys conducted with the 50 state coordinators and two major city coordinators between January and August 2001. The State Coordinator Survey (SCS) took approximately 40 minutes to complete with a copy of the survey having been mailed beforehand to facilitate preparation for the telephone interview. The New Jersey and Ohio state coordinators were also interviewed in person. Subsequent to the initial telephone and in-person interviews, additional contacts were made by telephone or email to request omitted information or to clarify previous answers. Face-to-face interviews were held with D.A.R.E. America staff.

### Instrument development

Items for the State Coordinator Survey were developed with reference to a number of sources, including a literature review, consultations with key leaders in substance abuse prevention (Drs. Herbert Kleber and Zili Sloboda) and officers and directors from D.A.R.E. America, interviews with two state coordinators (New Jersey and Ohio), and focus groups and interviews with D.A.R.E. officers. Interviews with the D.A.R.E. America personnel were conducted at the national D.A.R.E. offices and at the annual National D.A.R.E. Officers Association Conference in June, 2001. These informal, open-ended interviews provided the background and history of D.A.R.E. and insights into the diversity of the state organizations.

D.A.R.E. officers and several coordinators were also interviewed at the conference or by telephone and about 8–10 officers participated in each of two focus groups. Most of these officers had been active in the program for many years and about half the officers in each group were mentors. These interviews and discussions provided information about how local D.A.R.E. officers functioned within their agencies and in collaboration with schools. They also provided suggested areas to be pursued with state coordinators regarding the interrelationship between the state and local levels. Following from these various consultations, the State Coordinator Survey was developed. The final instrument was comprised of five sections, as follows:

1. Organization: Coordinator's roles; time involvement with D.A.R.E.; links to the D.A.R.E. Officer Association (DOA); other communication channels with officers; relationship between state program and D.A.R.E. America; role of emerging Charter organizations.

2. Funding: Major sources and purpose of funding (including allocation of money between the state and local programs); competition with other prevention programs; Fiscal Year 2000 state budgets (the latest year for which these data were universally available).

3. Training and Quality Control: Periodicity and kinds of trainings; quality control and monitoring of officers, including the role of both police and schools in maintaining program fidelity and quality; role of D.A.R.E. mentors/field representatives.

4. Community Efforts: Relationships with other programs; parent curriculum; parent involvement.

5. Feedback/Concerns: Suggestions from coordinators for modifying or improving the D.A.R.E. organization and the relationships between local, state and national organizational levels.

## Competing interests

Zili Sloboda is engaged in an ongoing study of a new school-based curriculum based in part on the existing D.A.R.E. program and supported by the Robert Wood Johnson Foundation.

## Authors' contributions

JM conceived of the study with ZS. JM participated in its design and coordination and helped to draft the manuscript. IP assisted in the overall conception of the study, participated in its design and coordination, performed data collection and helped to draft the manuscript. LAKJ performed the literature review, participated in data analysis, and performed substantial drafting and rewriting of the manuscript. TD participated in the design and coordination of the study, performed data collection, and literature review. ZS helped to draft the manuscript and wrote the discussion. All authors (except JM, who died prior to the final draft of the paper was completed) read and approved the final manuscript.

## References

[B1] Boles SM, Miotto K (2003). Substance abuse and violence: A review of the literature. Aggression and Violent Behavior.

[B2] Brook JS, Richter L, Rubenstone L (2000). Consequences of adolescent drug use on psychiatric disorders in early adulthood. Annals of Medicine.

[B3] Ellickson PL, Tucker JS, Klein DJ (2001). High-risk behaviors associated with early smoking: results from a 5-year follow-up. Journal of Adolescent Health.

[B4] Logan TK, Walker R, Cole J, Leukefeld C (2002). Victimization and substance abuse among women: contributing factors, interventions, and implications. Review of General Psychology.

[B5] Pentz MA, Bukoski WJ, Evans RI (1998). Costs, benefits, and cost-effectiveness of comprehensive drug abuse prevention. Cost-benefit/cost-effectiveness research of drug abuse prevention: Implications for programming and policy Monograph 176.

[B6] Robert Wood Johnson Foundation [RWJF] (2001). Substance abuse: the nation's number one health problem.

[B7] Substance Abuse and Mental Health Services Administration [SAMHSA] (2002). Mortality data from the Drug Abuse Warning Network.

[B8] CASA (2000). The National Center on Addiction and Substance Abuse: Shoveling up: The impact of substance abuse on state budgets.

[B9] Merrill J, Fox K, Bukowski WJ, Evans RI (1998). The impact of substance abuse on federal spending. Cost-benefit/cost-effectiveness research of drug abuse prevention: Implications for programming and policy Monograph 176.

[B10] National Institute on Drug Abuse [NIDA] (1992). The economic costs of alcohol and drug abuse in the United States – 1992.

[B11] Hallfors D, Watson K (1998). Organization of drug prevention services in the health care delivery system.

[B12] Pentz MA, Dwyer JH, MacKinnon DP, Flay BR, Hansen WB, Wang EY, Johnson CA (1989). A multi-community trial for primary prevention of adolescent drug abuse: Effects on drug use prevalence. JAMA.

[B13] Botvin GJ, Baker E, Dusenbury L, Botvin EM, Diaz T (1995). Long-term followup results of a randomized drug abuse prevention trial in a white middle-class population. JAMA.

[B14] Sloboda Z, David SL (1997). Preventing Drug Use Among Children and Adolescents: A Research-Based Guide, NIH Publication No.

[B15] Dusenbury L, Falco M, Lake A (1997). A review of the evaluation of 47 drug abuse prevention curricula available nationally. J Sch Health.

[B16] Petrosino A (2003). Standards of evidence and evidence for standards: The case of school-based drug prevention. Annals.

[B17] D.A.R.E. About D.A.R.E. http://www.DARE.com/InsideDAREAmerica.

[B18] General Accounting Office Youth Illicit Drug Use Prevention: DARE Long-Term Evaluations and Federal Efforts to Identify Effective Programs.

[B19] Johnston LD, O'Malley PM, Bachman JG, Schulenbeg JE (2005). Monitoring the Future national results on adolescent drug use: Overview of key findings, 2004. (NIH Publication No05–5726).

[B20] Hallfors D, Godette D (2002). Will the "Principles of Effectiveness" improve prevention practice? Early findings from a diffusion study. Health Educ Res.

[B21] Ringwalt CL, Geene JM, Ennett ST, Lachan R, Clayton RR, Leukefeld CG (1994). Past and future directions of the D.A.R.E. program: An evaluation review. http://www.ncjrs.org/txtfiles/DARErev.txt.

[B22] Ennett ST, Tobler NS, Ringwalt CL, Flewelling RL (1994). How effective is Drug Abuse Resistance Education? A meta-analysis of Project D.A.R.E. outcome evaluations. Am J Pub Health.

[B23] D.A.R.E http://www.DARE.com..

[B24] Merrill J, Dilascio T, Pinsky I (2002). Law enforcement and drug prevention: A profile of the D.A.R.E. officer. The Police Chief.

[B25] D.A.R.E. Form 990: Return of organization exempt from income tax, 2000. http://guidestar.org.

[B26] Elliot J (1995). Drug Prevention Placebo: How D.A.R.E. Wastes Time, Money, and Police. Reason.

[B27] Shephard E (2001). The economic costs of D.A.R.E. Institute of Industrial Relations, Research Paper Number 22.

[B28] Project Alert. http://www.projectalert.best.org.

[B29] Life Skills Training. http://www.lifeskillstraining.com.

